# Daily Light Onset and Plasma Membrane Tethers Regulate Mitochondria Redistribution within the Retinal Pigment Epithelium

**DOI:** 10.3390/cells13131100

**Published:** 2024-06-25

**Authors:** Matilde V. Neto, Giulia De Rossi, Bruce A. Berkowitz, Miguel C. Seabra, Philip J. Luthert, Clare E. Futter, Thomas Burgoyne

**Affiliations:** 1 UCL Institute of Ophthalmology, University College London, London EC1V 9EL, UK; 2 Department of Ophthalmology, Visual and Anatomical Sciences, Wayne State University School of Medicine, Detroit, MI 48202, USA; 3 iNOVA4Health, NOVA Medical School, Faculdade de Ciências Médicas, Universidade NOVA de Lisboa, 1169-056 Lisboa, Portugal

**Keywords:** retinal pigment epithelium, mitochondria, membrane contact sites

## Abstract

The retinal pigment epithelium (RPE) is an essential component of the retina that plays multiple roles required to support visual function. These include light onset- and circadian rhythm-dependent tasks, such as daily phagocytosis of photoreceptor outer segments. Mitochondria provide energy to the highly specialized and energy-dependent RPE. In this study, we examined the positioning of mitochondria and how this is influenced by the onset of light. We identified a population of mitochondria that are tethered to the basal plasma membrane pre- and post-light onset. Following light onset, mitochondria redistributed apically and interacted with melanosomes and phagosomes. In a choroideremia mouse model that has regions of the RPE with disrupted or lost infolding of the plasma membrane, the positionings of only the non-tethered mitochondria were affected. This provides evidence that the tethering of mitochondria to the plasma membrane plays an important role that is maintained under these disease conditions. Our work shows that there are subpopulations of RPE mitochondria based on their positioning after light onset. It is likely they play distinct roles in the RPE that are needed to fulfil the changing cellular demands throughout the day.

## 1. Introduction

The retinal pigment epithelium (RPE) is a monolayer of polarized epithelial cells which, in partnership with the Bruch’s membrane, forms the outer blood–retinal barrier, separating the retina from the blood supply of the adjacent choroid. The RPE has a myriad of functions to maintain retinal health including a central role in energy production, protecting against light radiation, controlling the subretinal space volume, and the transport of metabolites, ions, and water from the choriocapillaris to support the neuroretina [1].

The visual system is highly energy-demanding and defects in mitochondrial function within the RPE have been linked to diseases including age-related macular degeneration (AMD) [2]. There is evidence that AMD patients have fewer mitochondria with altered morphology and increased mitochondrial DNA damage [3,4,5]. Furthermore, a study involving the ablation of RPE mitochondrial oxidative phosphorylation in mice demonstrated photoreceptor degeneration [6]. These studies highlight the importance of mitochondria in the normal healthy function of the RPE. In addition to producing adenosine triphosphate (ATP), mitochondria are also known to participate in the scavenging of ROS, regulating calcium homeostasis, nuclear signaling, and the synthesis of amino acids, cholesterol, and phospholipids [7,8,9,10]. The spatial distribution of mitochondria within the cell influences energy production and delivery to specific subcellular regions where it is needed [11]. Therefore, mitochondrial movement and regions of close apposition between mitochondrial and other organelle membranes, known as membrane contact sites (MCSs), can likely aid in adapting and responding to changes in the local environment [12]. Previous studies including our work have shown that mammalian RPE is heavily populated with a heterogeneous population of mitochondria based on membrane potential and ATP production, and these mitochondria predominantly localize to the basal region of the cell [13,14,15]. 

Transport of glucose, Ca^2+^, Cl^−^, K^+^, and HCO^3−^ across the basal and apical membranes of the RPE involves GLUT1 transporters and a range of ion channels and transporters on the basal and plasma membranes [16,17]. This is optimized at the basal surface of the RPE by elaborate infoldings of the plasma membrane that act to increase the surface area. Morphological changes in these basal infoldings and reduced surface area have been described in models of aging and eye disease including choroideremia (CHM) [13,18,19]. Loss of function of Rab Escort Protein 1 (REP1) in CHM results in the reduced prenylation of Rab proteins, which are involved in multiple intracellular trafficking pathways [20]. This leads to progressive degeneration of the RPE, photoreceptors, and the choroid [21]. The RPE of CHM mouse models has reduced numbers of melanosomes in the apical processes and delayed phagosome degradation [19]. Phagocytosis is a circadian rhythm-dependent process and plays a vital role in the turnover of photoreceptor outer segments, which involves the daily uptake of the distal portion of the outer segments to allow degradation and recycling of proteins and lipids [22,23]. Under normal healthy conditions, re-isomerization of all-*trans*-retinal into 11-*cis*-retinal occurs and it is recycled back to the photoreceptors [24,25]. When this process is perturbed, it has implications for the homeostasis of the retina, including accumulation of toxic lipofuscin within the RPE. 

In this study, we performed an ultrastructural analysis to characterize the position of mitochondria within both human and mouse RPE cells, as well as their distribution after light onset. The results revealed MCSs where mitochondria are tethered to the basal surface of RPE cells. We also observed that while some mitochondria redistribute to the apical surface of RPE cells and make contact with melanosomes and phagosomes after light onset, a significant mitochondria pool remains anchored to the basal surface of the cells. This suggests the existence of two distinct mitochondria subpopulations; (i) one with a constitutive function performed by tethered basal mitochondria and (ii) one with a stimulated role performed by the untethered and motile mitochondria. In a Chm mouse model that had areas with altered or lost basal infoldings, we found that the tethering of mitochondria to the basal membrane was not affected. This provides evidence for the importance of mitochondria tethering to the basal plasma membrane in RPE cells. 

## 2. Materials and Methods

### 2.1. Human Tissue

Donor eye tissue from a 90-year-old with no known eye disease was approved for research and received from the Moran Eye Institute, Salt Lake City, UT, USA, courtesy of Dr GS Hageman.

### 2.2. Mouse Tissue

Two-month-old male wild-type C57BL6 mice were sacrificed before light onset (dark-adapted overnight), 1 hour after light onset, or 6 hours after light onset (3 eyes each from a separate mouse for each timepoint) and prepared from transmission electron microscopy, UK Home Office license number PPL: PP5562410. Eyes from the conditional knock-out mouse line *Chm^Flox^*, *Tyr-Cre* had been prepared for transmission electron microscopy in a previous study, under project licenses 70/6176 and 70/7078. The *Chm^Flox^*, *Tyr-Cre* line carries the *Cre* recombinase transgene, which is under the control of the tyrosinase promoter and generated previously as described [26]. All mice were sacrificed by cervical dislocation at a Schedule 1-approved designated establishment in accordance with the Animals (Scientific Procedures) Act 1986 (United Kingdom) and Home Office (United Kingdom) guidance rules, adhering to the Association for Research in Vision and Ophthalmology Statement for the Use of Animals in Ophthalmic and Vision Research.

### 2.3. Transmission Electron Microscopy

Mouse eyes were fixed in 2% PFA and 2% glutaraldehyde in 0.1 M cacodylate buffer for 1 h at room temperature, before removing the cornea and lens and fixing for a further 1 h at room temperature, as previously described [27]. The eyes were washed in 0.1 M cacodylate buffer and incubated in 1% osmium tetroxide and 1.5% potassium ferrocyanide in distilled water for 1 h in the dark at 4 °C. Subsequently, the eyes were dehydrated in increasing concentrations of ethanol (70%, 90%, and 100%) and in a mixture of propylene oxide:epon (1:1) overnight at room temperature. The propylene oxide:epon was replaced with two changes of epon every 3 h at room temperature before embedding in epon ovenight at 60 °C. Then, 100 nm sections were cut and stained with Reynolds’ lead citrate before imaging on a JEOL 1400Plus EM (JEOL ltd, Tokyo, Japan) fitted with both an Advanced Microscopy Technologies (AMT) NanoSprint12 (AMT Imaging Direct, Woburn, MA, USA) and a Gatan Orius SC1000B camera (Gatan, Pleasanton, CA, USA). Images were analyzed using ImageJ, including using a custom-written script to divide images into 3 equal-sized regions.

### 2.4. Electron Tomography

Tomography tilt series were collected over a range of ±60° with two perpendicular axes using electron microscopy sections loaded on a JEOL 1400Plus (JEOL ltd, Tokyo, Japan), along with the software SerialEM (version 4.0.28 developed at the University of Colorado, Boulder). Dual-axis tomograms were generated from the tilt data using IMOD (version 4.11 developed at the University of Colorado, Boulder) [28]. Image data were viewed in 3dmod (part of the IMOD package) and ImageJ (version 1.54f).

## 3. Results

### 3.1. Mitochondria Are Tethered to the Basal and Lateral Plasma Membrane of the RPE

**Figure 1:**
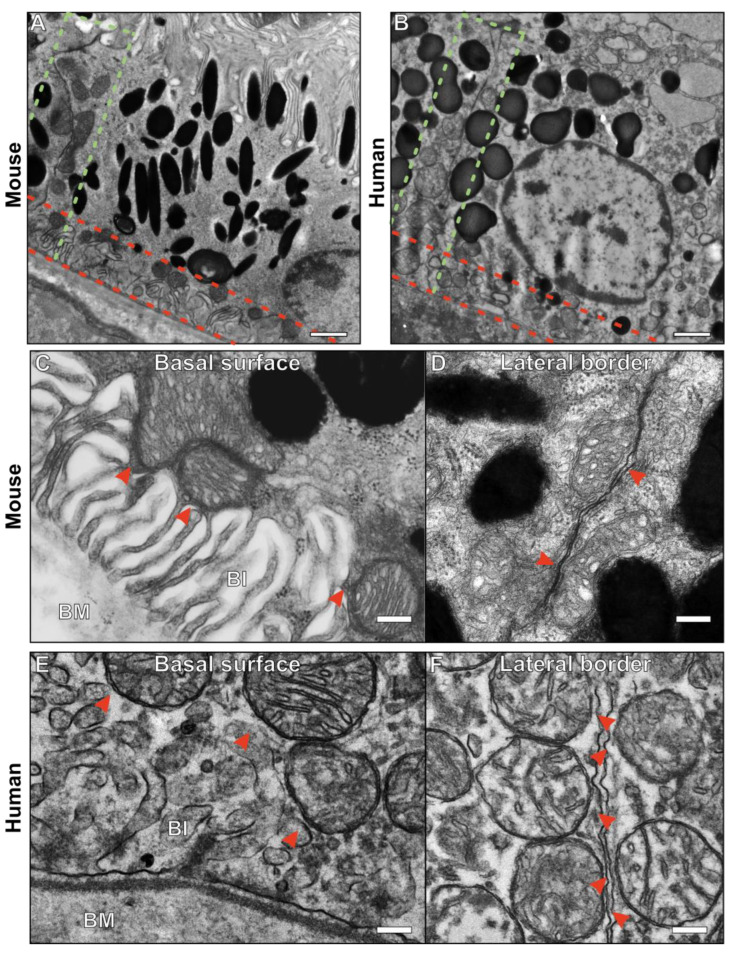
Mitochondria are in contact with the plasma membrane at the basal surface and lateral border of the RPE. (**A**,**B**) Electron microscopy images of mouse and human RPE with the lateral border shown between the green dotted lines and the basal region between the red dotted line. (**C**–**F**) Higher magnification images of the basal surface and lateral border of mouse and human RPE. (**C**,**E**) Mitochondria are in contact with the basal infoldings (BIs) of the plasma membrane as shown by the red arrowheads. The basal infoldings are intricate extensions of the plasma membrane surface that are positioned against the Bruch’s membrane (BM). (**D**,**F**) Mitochondria also make contact with the plasma membrane at the lateral border of the RPE as shown by the red arrowheads. Scale bars: (**A**,**B**) 1 µm, (**C**–**F**) 200 nm.

Many of the mitochondria within mouse and human RPE are positioned close to the plasma membrane (PM) at the lateral border between neighboring cells and at the basal surface (Figure 1). When looking at high magnification by electron microscopy, the intricate infolding of the PM at the basal surface can be seen to be in contact with mitochondria, with similar MCSs visible at the lateral borders between RPE cells (Figure 1A–E). The basally infolded PM in mouse RPE appears more extensive in the example shown in Figure 1C and has an ‘open’ appearance unlike that of the human basal infoldings (Figure 1E). At the lateral border, multiple mitochondria can be seen alongside the plasma membrane (Figure 1D,F).

**Figure 2:**
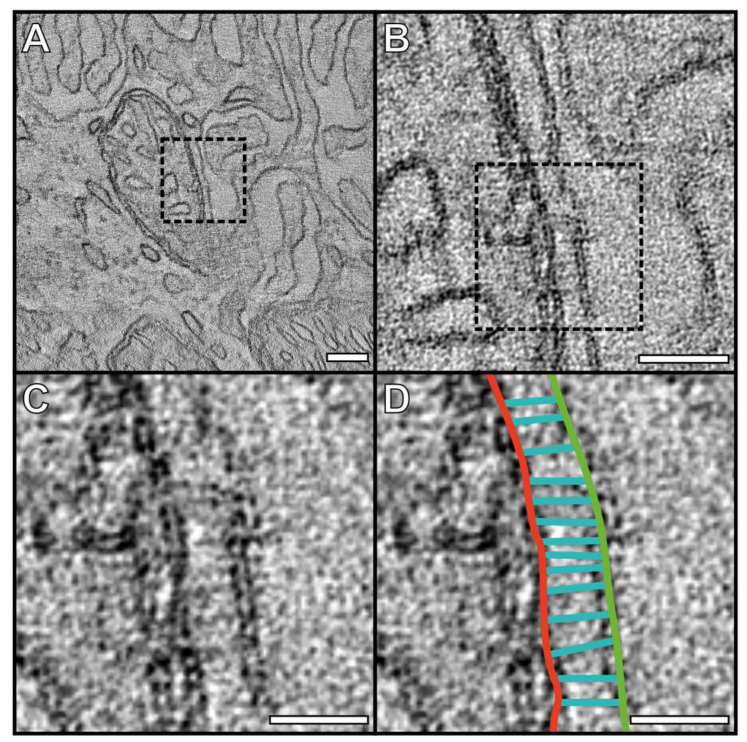
Electron tomography resolves tethers between the outer mitochondrial membrane and the plasma membrane at the basal surface of mouse RPE. (**A**) A slice from a tomogram with zoomed-in images shown in (**B**–**D**). (**D**) The outer mitochondrial membrane is shown in red, the plasma membrane in green, and the tethers in cyan. Scale bars: (**A**) 100 nm, (**B**) 50 nm, (**C**,**D**) 25 nm.

To characterize the MCSs between mitochondria and the PM of basal infoldings, tomograms were generated (Figure 2, Figure S1, Videos S1 and S2). As this technique provides higher-resolution imaging compared to conventional TEM, it is possible to resolve structures at the site between the mitochondria and PM. This includes fibrous-like tethers that can be seen running in between and adjoining the outer mitochondrial membrane to the PM.

### 3.2. After Light Onset, Mitochondria Redistribute from Basal to Apical RPE

**Figure 3:**
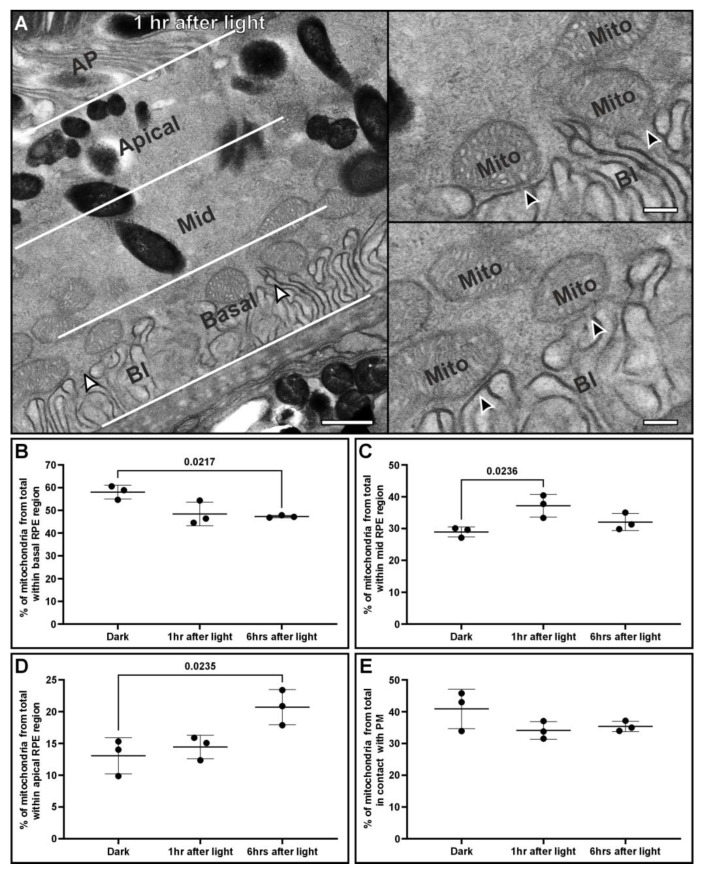
Mitochondria move from the basal surface to the apical side of the RPE after light onset. (**A**) Electron microscopy image from a mouse eye prepared 1 h after light onset, showing how the RPE can be split into three regions, basal, mid, and apical. These regions are positioned between the basal infolding (BI) and the base of the apical processes (APs) of the RPE. High-magnification panels of the regions indicated by the white arrowheads show the membrane contact sites between mitochondria and the infolded plasma membrane (black arrowheads) at the basal surface. (**B**–**D**) Measurements of the proportion of mitochondria within different regions of the RPE before and after lights (n = 3 eyes and >430 mitochondria analyzed per timepoint). After light onset, there is significant repositioning of mitochondria from the basal region through to the mid (at 1 h after light onset) and apical regions (6 h after light onset) of the RPE. (**E**) When looking at the proportion of mitochondria in contact with the basal surface of the RPE, there was no significant difference between the timepoints. Mean shown with the standard deviation and statistical significance was determined by one-way ANOVA and Tukey’s multiple comparisons test. Scale bars: (**A**) left panel 600 nm and right panels 200 nm.

The positions of mitochondria, including those in contact with the PM (<30 nm distance between the two), were assessed in mouse eyes prepared at different timepoints, including pre- and post-light onset (Figure 3). TEM images of the mouse RPE were split into three regions, apical, mid, and basal (Figure 3A), avoiding areas with the cell nucleus (due to its large size across multiple regions of the cell). The number of mitochondria was quantified in each region, as was the number of mitochondria attached to the basal infoldings (Figure 3B–E). No difference was found in the total number of mitochondria within RPE cells at the different timepoints (Figure S2A,B). Regions at the lateral membranes of the RPE were excluded from this analysis due to the same number of tethered mitochondria being positioned across them at all timepoints, indicating that they do not reflect mitochondrial repositioning (Figure S2A,C). After light onset, there was a redistribution of mitochondria from the basal region through to the mid (1 h after light onset) and then into the apical region (6 h after light onset) of the RPE (Figure 3B–D). While there were statistically significant changes in the proportion of mitochondria within different regions of the cells at different times of the day, there was no significant difference in the proportion of mitochondria in contact with the PM (Figure 3E). 

### 3.3. Redistribution of Mitochondria to the Apical Region Is Accompanied by Increased Interaction with Melanosomes and Phagosomes

**Figure 4:**
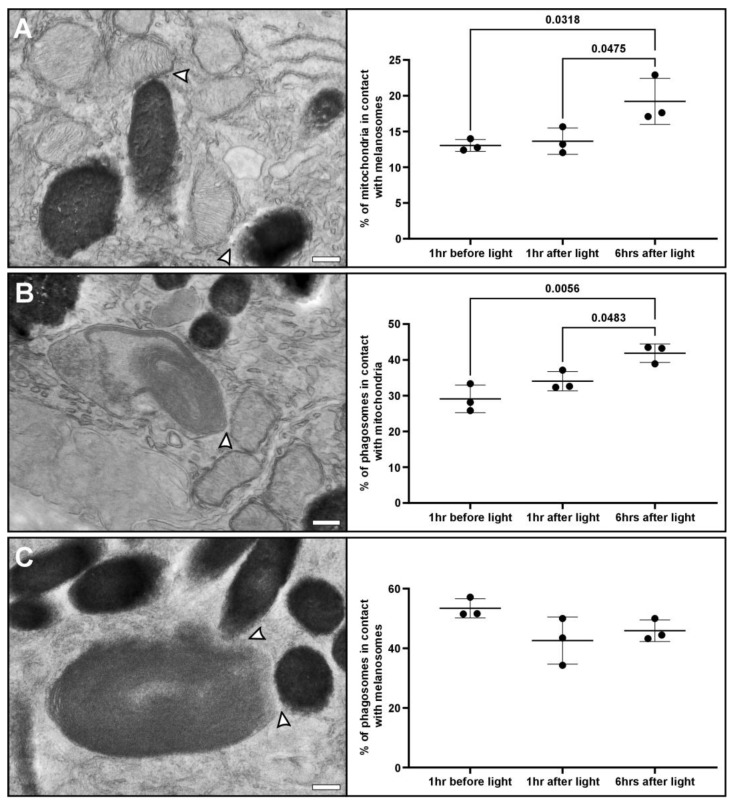
After light onset, there is increased interaction of mitochondria with melanosomes and phagosomes containing a photoreceptor outer segment (POS). Electron microscopy images showing membrane contact sites (white arrowheads) between (**A**) mitochondria and melanosomes, (**B**) POS and mitochondria, and (**C**) POS and melanosomes. At a timepoint of 6 h after light onset, there are significantly more mitochondria in contact with (**A**) melanosomes and (**B**) phagosomes compared to dark-adapted and 1 h after light onset. (**C**) There was no difference in the percentage of POS in contact with melanosomes at the different timepoints. n = 3 mouse eyes and >310 mitochondria or >110 POS were examined at each timepoint. Mean shown with the standard deviation and statistical significance was determined by one-way ANOVA and Tukey’s multiple comparisons test. Scale bar: 250 nm.

To investigate other types of MCSs at different timepoints, we examined interactions between mitochondria, melanosomes, and phagosomes containing a photoreceptor outer segment (POS). We found a significant increase in the proportion of mitochondria with MCSs with melanosomes and with phagosomes 6 h after light onset (Figure 4A,B). There was no significant difference in the number of phagosomes in contact with melanosomes (Figure 4C).

### 3.4. Mitochondrial Positioning, but Not the Tethering of Mitochondria to the Plasma Membrane, Is Affected in the RPE of Choroideremia Mice

**Figure 5:**
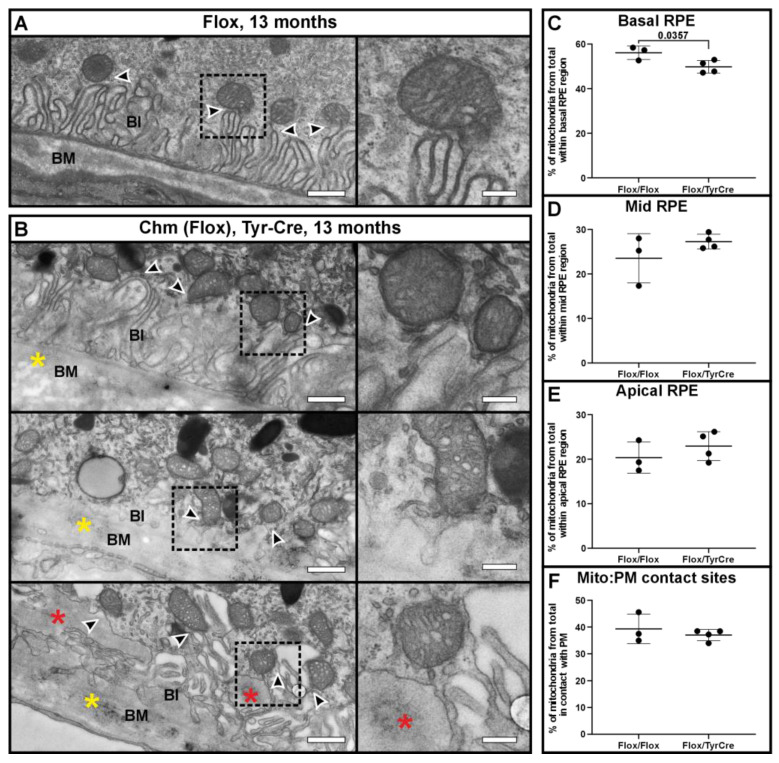
Mitochondrial–PM membrane contact sites are not disrupted at the basal RPE in a choroideremia mouse model. (**A**) Flox control mice present a normal morphology of the basal infoldings. (**B**) The RPE-specific choroideremia mouse model has regions with basal infoldings that are present (top panel), absent (middle panel), or have basal deposits (shown by red asterisks in the bottom panels). In this model, there is thickening of the Bruch’s membrane (BM), as highlighted by the yellow asterisk. (**A**,**B**) In both models, the mitochondria can be seen in contact with the basal plasma membrane, as shown by the black arrowheads. (**C**–**E**) When comparing the proportion of mitochondria within different regions of the RPE, there are significantly fewer mitochondria within the basal region of the cell. (**F**) Between the two models, there is no significant difference in the number of mitochondria in contact with the basal plasma membrane. n = 3 eyes and >650 mitochondria analyzed for each model. The eyes were taken 1 h after light onset. Mean shown with the standard deviation and statistical significance was determined by one-way ANOVA and Tukey’s multiple comparisons test. Scale bars: (**A**,**B**) righthand panels 600 nm and lefthand panels 200 nm.

A choroideremia mouse model was examined to determine if mitochondrial positioning and the contact sites with the basal PM were affected (Figure 5). The model used is cell-type specific, where the *Chm* gene is only knocked out in pigmented cells (*Chm^Flox^*, *Tyr-Cre+*). The Flox control and Chm mouse eyes were prepared at the same time of day, 1.5 h after light onset. Compared to the Flox control (Figure 5A), the Chm mice had regions with evident thickening of Bruch’s membrane, with basal deposits and disrupted basal infoldings as has been previously reported in this model [19] (shown in Figure 5B). A significant reduction in the proportion of mitochondria located in the basal regions of the RPE was found in the Chm mice (Figure 5C). This coincided with an increase in the proportion of mitochondria within the mid and apical regions of the RPE, despite it not being significant (Figure 5D,E). Even though there were fewer mitochondria in the basal region of the Chm mice, there was no significant difference in the proportion of mitochondria tethered to the PM when compared to total mitochondria. Regions of the RPE of the Chm mice that had disrupted or had no basal infoldings still had visible MCSs between mitochondria and the PM, as shown in Figure 5B.

## 4. Discussion

Mitochondria play an essential role in retinal health, and dysfunction within the RPE has been associated with a number of visual diseases. In this study, we show that there are two subpopulations of mitochondria within mouse RPE, ones that redistribute after light onset and a second group that is tethered to the basal plasma membrane. As the RPE is a highly specialized epithelium that performs a myriad of functions essential for retinal health, these two mitochondrial populations likely play distinct and critical roles needed for healthy vision.

Our previous work and that of others have shown a ‘carpet’-like arrangement of mitochondria positioned against the basal surface of mouse RPE in contact with the basal infoldings [13,15]. Nutrients, metabolites, solutes, and water are transported from the endothelial cells of the choriocapillaris across the basal infoldings into the RPE [29,30,31]. Therefore, the ‘carpet’ of mitochondria is well-placed to provide a large surface area that is directly in contact with the incoming resources. The mitochondria are also well situated to provide ATP to a number of ATP-dependent ion pumps and channels, including Na^+^/K^+^-ATPase, Ca^2+^-ATPase, Kir7.1, and Bestrophin 1 [17,32,33]. For this mitochondrial arrangement against the PM to exist and be maintained, they need to be held in place. Using electron tomography, we were able to resolve tethers that run between the mitochondrial outer membrane and the PM. These resemble the tethering of mitochondria to the PM in mouse photoreceptors that we previously identified [27]. This type of contact site has not been well studied in mammalian cells, and the composition of the tethers is unknown.

By looking before and after light onset, we were able to detect mitochondrial redistribution within the RPE. Throughout the day after light onset, we found mitochondria redistributed away from the basal region of the cell towards the apical surface. Mitochondrial movement within the RPE has been studied in cell culture models by fluorescence microscopy but has not been shown in tissues at the resolution of electron microscopy that allows precise determination of apical vs. basal distribution [14,34,35]. The fixative used in electron microscopy can cause some sample shrinkage, but generally good preservation is maintained, as has been shown in correlative live cell imaging and electron microscopy studies [36,37]. Surprisingly, when assessing the proportion of mitochondria tethered to the basal surface, there was no significant difference at the timepoints studied. It is possible that the non-tethered mitochondria within the basal region of the RPE do not move after light onset. Further work is needed to determine if this is true and if the MCSs between the mitochondria and the PM are dynamic, as it is possible that the mitochondria that move and those that are tethered interchange with time.

To better understand the rationale for mitochondrial redistribution within the RPE towards the apical surface after light onset, we investigated other types of MCS, including those between mitochondria, melanosomes, and phagosomes. Interactions between mitochondria and melanosomes have been described in melanocytes and it is believed that this is due to melanosomes acting as reactive oxygen species (ROS) scavengers [38]. Throughout the day, due to the metabolic activity, ROS accumulates in mitochondria, and the interaction with melanosomes will help buffer and reduce the harmful effects of this ROS. It has been suggested that aging can lead to overproduction of ROS that damage mitochondria and this can be a contributing factor to AMD [39,40]. Therefore, it is possible that the interaction with melanosomes is needed to maintain healthy mitochondria within the RPE. 

After light onset, we saw an increase in the interaction between mitochondria and phagocytosed POS. Phagosomes contain a mixture of saturated and highly unsaturated fatty acids that can provide the RPE with substrates for mitochondrial and peroxisomal fatty acid β-oxidation [41]. The interactions between mitochondria and phagosomes likely form part of the mechanism to recycle metabolic intermediates back to the outer retina. As part of the circadian cycle, after light onset, it is known that there is a burst of photoreceptor outer segment phagocytosis into the apical region of the RPE [22,23]. Mitochondrial redistribution to the apical region of the RPE would facilitate interaction with phagosomes. During phagosome maturation, phagosomes move from the apical region to the basal region of the RPE and this would likely contribute to an increase in interactions between them and mitochondria [42].

We did not see a difference in the interaction between melanosomes and phagosomes at the different timepoints we observed. For each timepoint we found over 40% of phagosomes in contact with melanosomes, indicating that these interactions persist throughout the day. In contrast, the mitochondrial interactions with melanosomes and phagosomes appear to be dependent on their position within the RPE. It has been indicated that the interactions between phagosomes and melanosomes allow melanosomes to scavenge ROS and melanin to assist in POS disc turnover [43]. Future studies could benefit from ex vivo samples to help identify regulators and better understand the role of these different types of mitochondrial membrane contact sites.

In a choroideremia (Chm) mouse model, regions of the RPE basal plasma membrane were clearly affected as previously described, including abnormal morphology and regions with complete loss of infoldings or basal lipid deposits [19]. When comparing mitochondrial positioning between Chm mice and Flox controls, there were fewer mitochondria found within the basal region of the cell but, interestingly, there was no significant difference in the proportion of mitochondria tethered to the basal plasma membrane. In regions that have abnormal or absent basal infoldings, it was evident that mitochondria were still tethered to the PM, emphasizing the importance of this tethering that is maintained even under the disease conditions found in choroideremia. The impact of the abnormal mitochondrial positioning in the Chm mice on the disease process is unknown but may result directly from reduced Rab GTPase prenylation, as Rab32 has been shown to be involved in mitochondrial dynamics [44,45]. This, together with the delayed phagosome processing and altered melanosome positioning in Chm mice, may impact mitochondrial interactions with these organelles throughout the day [19]. Identifying the tethers and regulators of the different types of mitochondrial membrane contact sites with the plasma membrane, melanosomes, and phagosomes will allow the impact of the loss of REP-1 on their formation and function to be tested.

This study sheds light on different populations of mitochondria within the RPE based on their redistribution and interaction with the PM, phagosomes, and melanosomes. The redistribution of mitochondria and their MCSs are likely to have implications for eye disease, making them an important area to study. Further work is required to learn more about the regulators of these mitochondrial MCSs, their functions, and their implications for disease.

## Data Availability

Data are contained within the article or Supplemental Materials.

## Supplementary Materials

The following supporting information can be downloaded at: https://www.mdpi.com/article/10.3390/cells13131100/s1. Figure S1: A further tomogram showing tethers between the outer mitochondrial membrane and the plasma membrane at the basal surface of mouse RPE. Figure S2: Analysis showing that the total number of mitochondria and those associated with the lateral membrane of the RPE do not change at different times of the day. Video S1: Video of the tomogram shown in Figure 2. Video S2: Video of the tomogram shown in Figure S1.
