# Supplementary material for: Daily Light Onset and Plasma Membrane Tethers Regulate Mitochondria Redistribution within the Retinal Pigment Epithelium
Source: Cells. 2024 Jun 25;13(13):1100. doi: 10.3390/cells13131100 (PMC11240580; doi:10.3390/cells13131100)
Supplement: Supplementary file 1 [file cells-13-01100-s001.zip › Supplementary Figures updated.pdf]

Circadian rhythm and plasma membrane tethers regulate mitochondria redistribution within the retinal pigment epithelium

Matilde V. Neto, Giulia De Rossi, Bruce A. Berkowitz, Miguel C. Seabra, Philip J. Luthert, Clare E. Futter and Thomas Burgoyne

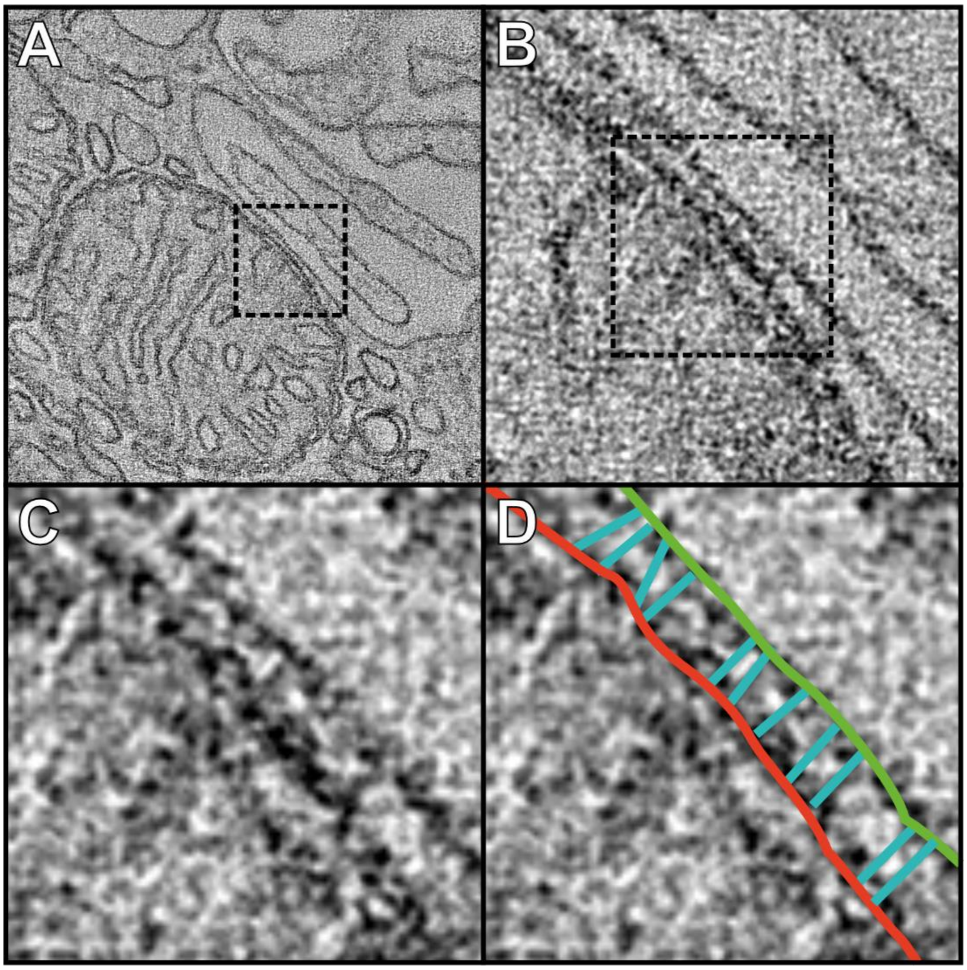

Figure S1. A further tomogram showing tethers between the outer mitochondrial membrane and the plasma membrane at the basal surface of mouse RPE. (A) A slice from a tomogram with zoomed in images shown in (B – D). (D) The outer mitochondrial membrane is shown in red, the plasma membrane in green and the tethers in cyan. Scale bars: (A) 100 nm (B) 50 nm (C & D) 25nm.

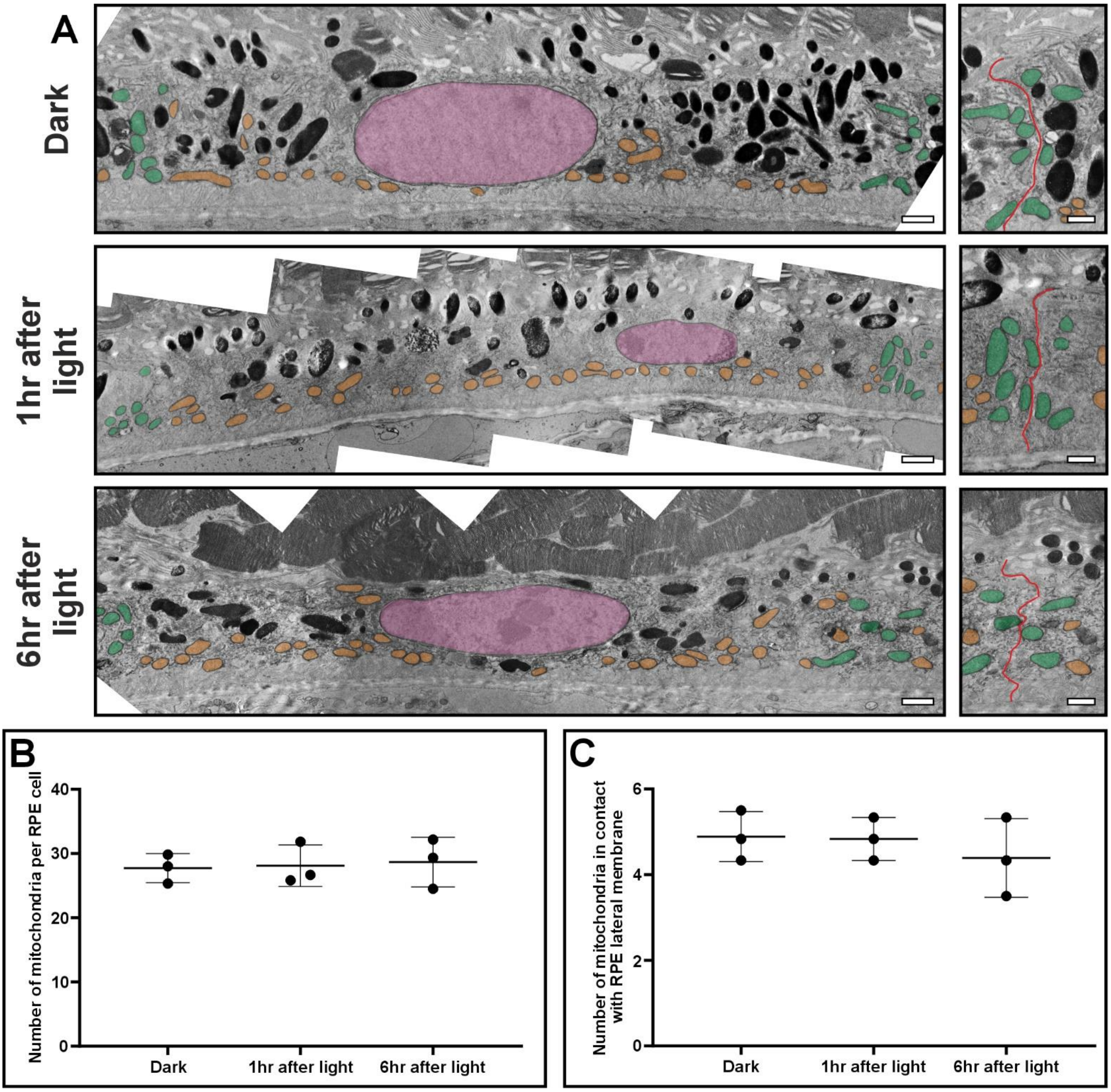

Figure S2. The total number of mitochondria and those associated with the lateral membrane of the RPE do not change at different time of the day. (A) Stitched electron microscopy images of RPE cells with the mitochondria false coloured green when within 500nm of the lateral junction or orange when away from the junctions. The cell nucleus is false coloured in purple. The panels on the righthand side show a zoomed in region at the lateral junctions from the whole RPE cells shown in the images on the lefthand side. The lateral membrane between RPE cells is false coloured in red in the righthand panels. By comparing electron microscopy images of the RPE from mouse eye prepared before and after light onset (B) the total number of mitochondria and (C) the number of mitochondria in contact with the lateral membrane were measured. N=3 mouse eyes and 18 RPE cells were analysed at each timepoint. Only cells that included a nucleus were examined to make sure that were all from approximately the same region of the cells. Graphs show the mean value and error bars are the standard deviation. Statistical significance was tested by one-way ANOVA and Tukey's multiple comparisons test. Scale bars: (A) left panels 1μm (B) right panels 500nm
